# Effective Conservative Management of Severe Scoliosis in a Girl with Prader–Willi Syndrome: A 20-Year Case Study Follow-Up

**DOI:** 10.3390/jcm14207350

**Published:** 2025-10-17

**Authors:** Calogero Malfitano, Francesco Negrini, Valentina Palloni, Marcello Meggiolaro, Elena Brevi, Piero Benfatti, Fabio Zaina, Giorgio Ferriero, Stefano Negrini

**Affiliations:** 1 Department of Biomedical Sciences for Health, University of Milan, 20133 Milan, Italy; calogero.malfitano@unimi.it; 2 Azienda di Servizi alla Persona Istituti Milanesi Martinitt e Stelline e Pio Albergo Trivulzio, 20146 Milan, Italy; 3 Physical and Rehabilitation Medicine Unit, Scientific Institute of Tradate, Istituti Clinici Scientifici Maugeri IRCCS, 21049 Tradate, Italy; giorgio.ferriero@uninsubria.it; 4 Department of Biotechnology and Life Sciences, University of Insubria, 21100 Varese, Italy; 5 Physical Medicine and Rehabilitation Residency Program, University of Milan, 20122 Milan, Italy; valentina.palloni@unimi.it (V.P.); marcello.meggiolaro@unimi.it (M.M.); elena.brevi@unimi.it (E.B.); 6 Retired General Practitioner, 63100 Ascoli Piceno, Italy; pbenfat@gmail.com; 7 ISICO (Italian Scientific Spine Institute), 20141 Milan, Italy; fabio.zaina@isico.it; 8 IRCCS Galeazzi—Sant’Ambrogio Hospital, 20157 Milan, Italy; stefano.negrini@unimi.it; 9 Department of Biomedical, Surgical and Dental Sciences, University of Milan, 20122 Milan, Italy

**Keywords:** conservative treatment, orthotic device, braces, spinal curvatures, treatment outcome, rehabilitation

## Abstract

**Background**: Prader–Willi syndrome (PWS) is a rare syndrome that presents in about 1 in 25,000 newborns. It is characterized by a typical phenotype that includes short stature, hypothyroidism and hypogonadism, cognitive and developmental delays, slow growth, obesity, and, in most patients, scoliosis. These patients generally have a life expectancy of less than 60 years, with respiratory distress being the leading cause of death; scoliosis is not the primary cause of these respiratory problems, but may contribute to their worsening. Therefore, accurately diagnosing and managing scoliosis is crucial for improving the life expectancy of PWS patients. Previous studies have shown a limited effectiveness of bracing due to a combination of factors, including generalized hypotonia, rapid early progression, poor brace compliance, and thus frequent progression to surgical intervention. **Case presentation**: This case report presents a 20-year follow-up of a female patient with PWS. Multiple clinical parameters were collected at every follow-up appointment. Throughout this extended observation and treatment period, the patient used two push-up braces of different rigidity, resulting in improvements in the thoracic and lumbar Cobb angle and the thoracic angle of trunk rotation. The treatment protocol was based on a shared decision with the parents and the patient. **Conclusions**: This case demonstrates how consistent and thorough follow-up can result in a successful, conservative treatment of a severe secondary scoliosis, thereby preventing the need for a major surgical procedure during growth.

## 1. Introduction

Prader–Willi syndrome (PWS) is a rare genetic disorder that occurs in approximately 1 in 25,000 newborns, most commonly resulting from a deletion of paternal genes on chromosome 15 [1,2]. Clinical features of PWS, especially in children lacking hormonal treatment, include short stature, small hands and feet, ligamentous hyperlaxity, developmental and cognitive delays, slow growth, low levels of thyroid and sex hormones, mild dysmorphic face, and impulsive attitudes like hyperphagia that can lead to obesity [1,2,3]. Scoliosis is also a common manifestation in PWS, with prevalence ranging from 15% to 86% [4,5,6]. The lifetime risk is estimated to be about 70% or higher until skeletal maturity [4,7,8]. De Lind et al. [9] observed that scoliosis in PWS exhibits a bimodal age distribution: infantile onset in 23% of patients, typically developing before age 4 and likely caused by early hypotonia, and the second peak during adolescence, when a curve starts in 45% of cases. People affected by PWS have a reduced life expectancy [10]; mortality is mainly related to respiratory disorders. These disorders can be worsened by scoliotic deformities [11,12]. Therefore, early diagnosis and treatment of scoliosis are imperative to improve patients’ quality of life and life expectancy.

Traditionally, scoliosis in PWS has been considered a complex condition to manage non-surgically, due to a combination of factors including generalized hypotonia, rapid early progression, poor brace compliance, cognitive and behavioral difficulties, and the mechanical challenges posed by obesity [7,13]. Thus, the previous literature has often described conservative treatment as minimally effective, with several authors reporting high rates of curve progression and eventual surgical indication [9,14]. On the other hand, surgical interventions, typically involving posterior spinal fusion, carry substantial neurological risk, as they are frequently performed on severe curvatures in individuals with a vulnerable spinal cord [14,15]. Moreover, if surgery is performed during growth, repeated procedures are frequently necessary [16]. Even if not resolutive and with some specific limits, bracing has been considered as a treatment able to postpone surgery in time [17].

This paper describes the case of a 24-year-old patient with PWS and scoliosis who has been under follow-up for 20 years. Due to the typical early onset and characteristic natural history of scoliosis in individuals with PWS, surgical treatment was the first recommended. Nevertheless, she began a brace treatment, combined with scoliosis-specific exercises (SSE), with the aim of postponing as much as possible the surgical procedure. The treatment plan was based on a shared decision-making process with her parents initially, and later with the patient herself, aiming to ensure the best possible outcome while maintaining an acceptable quality of life. The study is reported following the CARE guidelines [18]. Informed consent was obtained from the patient and the parents for publication.

## 2. Case Presentation

**Figure 1:**
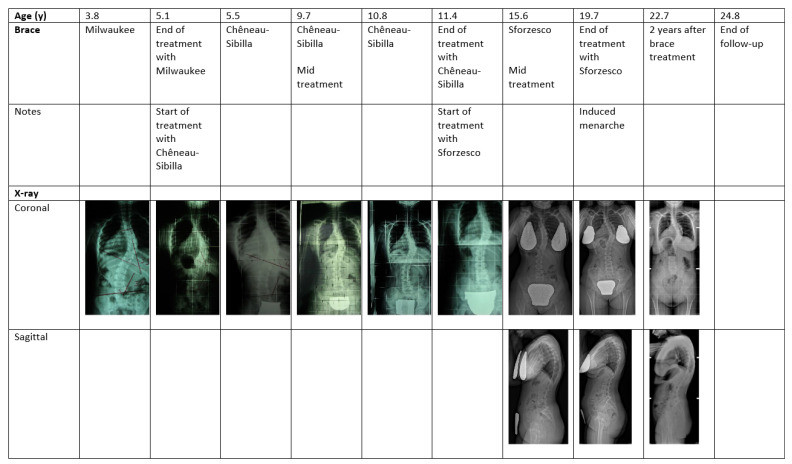

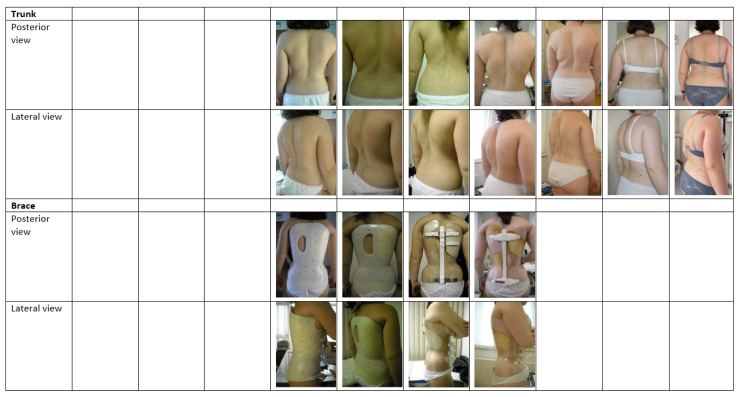
Radiographs and trunk and brace pictures at key treatment points in a girl with a scoliosis secondary to Prader–Willi syndrome. We began taking systematic photographs of cases in 2006; therefore, we missed the first picture of the girl. Brace refers to the type of brace used in the period preceding the evaluation when the photographs were taken.

The patient was diagnosed with PWS at the age of 3 years. Pregnancy was uneventful, and delivery occurred at term via Caesarean section due to neonatal distress. While verbal language development followed a typical timeline, walking acquisition and tooth eruption were delayed. Given that these delays could not be attributed to the neonatal distress, genetic testing was performed, leading to the diagnosis of PWS. Growth hormone (GH) therapy was initiated immediately after diagnosis (at 3.1 years), resulting in a rapid improvement in motor performance and the acquisition of independent walking. Upon achieving an upright posture, her parents noticed signs of scoliosis. At the age of 3.8 years, spinal radiographs confirmed moderate scoliosis, showing a right thoracic curve (D5–D11) of 39° Cobb and a left lumbar curve (D11–L4) of 42° Cobb (Figure 1).

She was initially evaluated at another facility, where surgery was considered the primary option. Meanwhile, a Milwaukee brace was prescribed for 18 h per day to control the curve until she reached an appropriate age for surgery. However, compliance was poor, and the scoliosis rapidly progressed.

At the age of 5.1 years, the patient was referred to our center. At the initial consultation, she presented with severe scoliosis, characterized by a right thoracic curve (D5–D11) measuring 50° Cobb and a left lumbar curve (D11–L4) measuring 34° Cobb. Clinically, the thoracic angle of trunk rotation (ATR) was 15°, and hump height measured 18 mm. No leg length discrepancy was observed. A shared decision between the medical team and the family to adopt conservative treatment was made. This decision aimed to postpone or possibly avoid surgery before bone maturity (palliation), and even to reduce the curve to prevent problems in adulthood. Given the poor tolerance to the Milwaukee brace, known to be associated with a higher risk of psychological discomfort [19], a push-up brace was subsequently prescribed [20]. Due to the young age, which did not require strong forces to be applied, the rigid version (Chêneau–Sibilla brace: rigid, push-up action, three-dimensional, monocot, ventral closure [20]) was chosen. Considering the severity of the curve, the maximum possible wearing time of 23 h per day was prescribed. The entire course of treatment was planned in agreement with previously published literature on the topic of rehabilitative treatment for scoliosis, specifically juvenile scoliosis [21,22]. To support adherence, the patient and caregivers were provided with printed educational materials and video resources available on a dedicated website regarding brace treatment. The family also had continuous access to the secretariat of the reference center, which was informed about the administrative procedures related to the provision of braces and SSE, ensuring comprehensive clinical and organizational support throughout the treatment period.

The patient was followed until the completion of skeletal growth with clinical evaluations every six months and annual radiographic assessments. After growth was completed, both clinical and radiographic evaluations were conducted yearly. Specifically, clinical assessment included: (1) anthropometric data (e.g., weight, height, and leg length discrepancy); (2) prominence measurements, namely the ATR (in degrees) and hump height (in millimeters) [23]; (3) the TRACE (Trunk Aesthetic Clinical Evaluation) Index, a validated instrument for evaluating the aesthetic appearance of the trunk asymmetry [24]. At each consultation, data were collected regarding daily brace usage hours, type, and frequency of SSE and sports activities. Radiological evaluation included anteroposterior spinal radiographs to obtain measurements of the Cobb angle and Risser grade, as well as lateral spinal radiographs to assess thoracic kyphosis, lumbar lordosis, and global balance.

After one year, the curve improved by 6° at the thoracic level and by 7° at the lumbar level, while the thoracic ATR decreased to 9° and the hump height to 9 mm. Therefore, the Chêneau–Sibilla brace, worn for 23 h per day, was confirmed as a treatment option and was kept in place until the age of 11 (Table 1).

At that point, a radiographic evaluation showed an improvement from the 50° at presentation: the thoracic curve was 26° and the lumbar curve was 18°, with a thoracic ATR of 5° and a hump of 11 mm. The progressive stiffening of the curve and progression due to puberty required transitioning from the rigid Chêneau–Sibilla to the super-rigid version of the push-up braces (Sforzesco: very rigid, push-up action, three-dimensional, bivalve, ventral closure [20]) to provide greater mechanical stiffness and improved corrective forces. The Sforzesco brace was prescribed 23 h per day until the patient reached 15 years of age, followed by a gradual weaning process. Upon reaching skeletal maturity between 17 and 18 years (Risser score > 3), the Cobb angle measured 45°, remaining below the commonly accepted surgical threshold and showing slight improvement from baseline. On the sagittal plane, a progression of hyperkyphosis was documented, with the kyphotic angle increasing to a maximum of 67° at approximately 15 years of age, and subsequently stabilizing within a range of 45–48° at the attainment of skeletal maturity. At the age of 20, the patient discontinued wearing the brace. Over the following three years without bracing, the thoracic Cobb angle stabilized at 53°, the lumbar curve at 27°, with thoracic ATR of 14°, and hump height of 25 mm. The sagittal plane showed a kyphotic Cobb angle fluctuating between 45° and 58°, with the sagittal index ranging from 65 to 75 mm. The TRACE index throughout the entire period showed a fluctuating but generally decreasing trend, indicating an overall improvement in trunk aesthetics over time. Initially, the scores were relatively high (up to 51.3 ± 7.7%), reflecting significant asymmetry. During brace treatment, the value gradually decreased to as low as 0 ± 20.2%, indicating minimal visible asymmetry, and then increased again as the brace was weaned off in adulthood. The reported compliance was fully satisfactory during the long treatment period for brace-wearing and SSE (Table 1).

The experience of the father was also collected: “From the very beginning, my daughter faced the brace with extraordinary patience, wearing it almost 24 h per day until the age of twenty. Even after our divorce, her mother and I remained united in supporting her, knowing that without conservative treatment, she would likely have needed multiple surgeries. Today, with her scoliosis stabilized, I feel grateful that her determination, together with the doctors’ expertise, made possible an outcome that once seemed out of reach.”

## 3. Discussion

PWS is known to present with multiple musculoskeletal complications, among which scoliosis is particularly frequent, with a lifetime prevalence estimated at approximately 70% or higher until skeletal maturity [4]. The natural history of scoliosis in PWS is often progressive and, along with muscle weakness, muscle hypotonia, and obesity, can severely impair respiratory function, significantly contributing to morbidity and mortality in this population [25]. Consequently, early identification and effective management of spinal deformities are crucial in enhancing patient outcomes and prolonging life expectancy. The current literature provides limited data on the outcomes of conservative treatment in patients with PWS. Andaloro et al. [14] noted that conservative management yielded suboptimal results, with 6 out of 11 braced patients experiencing progression of the spinal curve in the coronal plane. Nonetheless, sagittal alignment remained generally preserved, as most individuals maintained satisfactory thoracic kyphosis. The study highlighted poor patients’ compliance as a significant limitation, primarily attributed to the cognitive impairments commonly associated with PWS [14]. Additionally, brace design and fitting were complicated by patients’ obesity, with frequent adjustments required due to fluctuating body weight [14]. Therefore, they suggest that surgery remains the gold standard, even though it has a high rate of complications [14]. Likewise, van Bosse and Butler [4] reported that at least two patients experienced significant improvement in their curve with brace treatment. They emphasized the importance of close follow-up, even after skeletal maturity, to maintain long-term stability and monitor for potential deterioration, as surgery becomes likely with a curve exceeding 50°. Overall, in that cohort of patients, 15% of cases required surgical intervention [4,7]. Our results suggest that when bracing is implemented within a multidisciplinary framework, with careful attention to early detection, individualized orthotic design, frequent follow-up, and family education and SSE, the conservative treatment can be a viable and durable strategy. Although it is difficult to disentangle the respective contributions of bracing and SSE in curve stabilization, it is theoretically plausible that exercises could have supported postural control and trunk stability. Nonetheless, in this case, the patient’s cognitive profile may have reduced the capacity to correctly perform the exercises, thus limiting their potential effect and possibly making the role of bracing even more important. It is also important to note that positive results were achieved in this case, despite the patient reaching BMI values in the overweight range (≈26–28 kg/m^2^ in late adolescence), a condition that is usually considered a limiting factor for the effectiveness of bracing. This approach aligns with the current literature, which emphasizes the importance of rehabilitation as the first-line management for mild to moderate scoliosis, especially in syndromic cases where surgical risks are elevated [25]. Although surgical correction may be necessary in severe or rapidly progressive curves, many authors advocate for a cautious approach, reserving surgery for cases where bracing fails or respiratory compromise worsens [15].

The role of GH treatment in PWS remains debated: while it improves growth, body composition, and motor function, some studies suggest a possible association with the onset or progression of scoliosis, whereas others report no adverse effects, especially in younger children. In our case, GH therapy was started at the age of 3.1 years, with rapid improvement in motor function. However, because GH accelerates growth, careful monitoring is essential, as it may also favor a faster worsening of scoliosis. It is also possible that GH therapy anticipated the presentation of scoliosis; nevertheless, the vigilant monitoring by the parents allowed for early recognition and timely intervention, which likely contributed to the favorable long-term results obtained [26].

Scoliosis in PWS displays a progression pattern that is uniquely intermediate between adolescent idiopathic scoliosis and scoliosis secondary to neuromuscular disorders. Though not classically neuromuscular, its association with generalized hypotonia, motor delay, and postural abnormalities justifies its classification within syndromic scoliosis with neuromuscular components [4]. The use of different brace types tailored to the patient’s growth and curve progression, as documented here (Figure 1), is consistent with strategies reported in the literature for managing idiopathic and syndromic scoliosis, providing three-dimensional correction and improving axial trunk rotation [4,27]. Our findings of improved thoracic and lumbar Cobb angles and axial trunk rotation support these reports, highlighting that consistent bracing can stabilize or even reduce deformity in PWS-associated scoliosis. Moreover, compliance remains a key factor influencing the efficacy of bracing [28]. The patient’s initial partial compliance with the Milwaukee brace, followed by improved adherence with subsequent braces, underscores the importance of patient and family engagement, as well as the value of tailored treatment plans. This is particularly relevant in PWS, where cognitive and behavioral challenges can hinder treatment adherence [1]. It should also be noted that in this case, compliance data were based solely on self-reports, as objective monitoring with sensors had not yet been implemented in our clinical practice at the beginning of treatment. This represents a limitation, as patients are often not entirely accurate in reporting their time spent wearing braces. Nevertheless, the outcomes obtained in this case would be difficult to explain without a high level of adherence to the prescribed wearing schedule.

The stabilization of the spinal curve around 50° over a 20-year follow-up period effectively allowed us to defer surgical intervention. While the final Cobb angle remains at the lower threshold for surgical indication, the conservative approach successfully prevented the need for multiple procedures during the growth period. It is important to note that according to the Iowa natural history studies by Weinstein [29], curves exceeding 50° tend to progress more severely over time. However, these data refer to untreated patients, and we have less certainty regarding the long-term natural history of patients undergoing comprehensive rehabilitation treatment. Nevertheless, the slight progression observed in adulthood in our case represents a negative prognostic signal that requires the utmost attention. Future surgical consideration may still be needed depending on the long-term stability of the curve or the emergence of related complications. However, to date, surgery has been avoided due to the absence of both curve progression and clinical symptoms. Notably, the patient did not report any pain or respiratory compromise throughout the entire follow-up period. Continued clinical and radiographic monitoring is planned, and any future changes in health status will be documented and reported accordingly.

## 4. Conclusions

Our case report illustrates a successful long-term conservative treatment of scoliosis in a PWS patient, using a combination of orthotic braces over a 20-year follow-up period. Starting from a condition of severe scoliosis, initially considered surgical, it was possible not only to maintain the curvature under control (palliation) but also to achieve a reduction in its magnitude over time. This outcome was sustained until skeletal maturity, ultimately allowing the patient to avoid the surgical intervention that had previously been planned. Unfortunately, during the later stages of follow-up, the patient appeared to return to the initial condition, losing the previously achieved correction and maintaining only a stabilization of the curve. Ongoing observation will be necessary to monitor possible future changes in the clinical situation.

This case highlights the potential of a well-organized, long-term conservative approach to managing scoliosis in PWS. It suggests that, under specific conditions, surgery may be avoided or postponed. Most importantly, it underscores the need for further research to establish standardized protocols and to support clinical decision-making in the conservative treatment of scoliosis in PWS patients.

## Figures and Tables

**Table 1:** Clinical and radiographic data of the girl with scoliosis secondary to Prader–Willi syndrome from the first to the last consultation.

**Demographic Data**																					
Age (y)	3.8	5.1	5.5	6.3	7.8	8.7	9.7	10.8	11.4	12.4	13.1	13.8	14.8	15.6	17.0	17.9	18.3	19.7	20.8	22.7	24.8
Height (cm)		102	106	110	121	125	132.5	137.5	140	144	148	150	150	151.5	150	151	150	150.5	151.5	151	151
Weight (kg)		21	21	24	30	32.5	37.5	41.5	42.5	46.5	48	50	53.5	58	58.5	58.5	59.5	60.5	62.5	61	63.5
**Treatment**																					
Brace (hours per day)		MB (18)	CSB (23)	CSB (23)	CSB (23)	CSB (23)	CSB (22)	CSB (20)	CSB (20)	SB (22)	SB (20)	SB (21)	SB (20)	SB (18)	SB (16)	SB (14)	SB (12)	SB (8)	No	No	No
SSE: Times per week (minutes)		4 (45)	2 (45)	2 (45)	2 (45)			2 (45)		2 (45)	2 (45)		3 (30)	2 (30)	2 (45)	2 (45)	2 (45)	2 (45)		2 (45)	
Sport (times/week)						swim (2)	swim (2)	swim (2)		swim (2)			swim (2)	swim (2)	TR (2)	TR (2)	TR (2)	TR (5)	TR (2)	dance (1)	FB (2)
Notes	GH therapy ongoing from 3.1										Stop GH therapy							Induced menarche			
**X-ray**																					
Right thoracic curve (°)	39	50	44	37	32	32	41	35	26	34	40	41	40	43	45	45	50	50	48	53	
Left lumbar curve (°)	42	34	27	19	20	22	26	23	18	18	22	21	20	25	25	19	24	24	22	27	
Thoracic kyphotic (°)				40								21	67	53	59	46	48	45		58	
Lumbar lordosis (°)				36								42	53	49	55	51	53	53		58	
Sacral slope (°)				38								42	28	31	39	41	38	32		25	
Pelvic tilt (°)												8	25	22	19	18	19	32		25	
Pelvic incidence (°)												50	53	53	58	59	57	51		58	
Risser		0	0	0	0	0	0	0	0	0	0	0	0	0	2	4	4	4	4	5	
**Clinical data**																					
Right thoracic ATR (°)		15	9	12	12	9	10	10	5	7	8	6	6	7	12	9	10	10	12	14	14
Right thoracic Hump height (mm)		18	9	15	17	12	13	18	11	11	14	12	11	13	13	17	18	18	23	25	25
TRACE ± SD (%)		51.3 ±7.7	45.6 ±7.9	51.3 ±7.7	39.5 ±8.3	0±20.2	32.8 ±8.7	25.1 ±9.6	45.6 ±7.9	0±20.2	25.1 ±9.6	0±20.2	25.1 ±9.6	32.8 ±8.7	39.5 ±8.3	39.5 ±8.3	56.8 ±7.8	45.6 ±7.9	45.6 ±7.9	51.3 ±7.7	51.3 ±7.7
C7 plumbline (mm)		25	40	30	30	35	25	25	45	15	50	30	35	25	10	40	40	35	45	40	30
L3 plumbline (mm)		0	30	25	35	35	40	25	30	20	20	35	25	25	35	30	60	30	30	25	40
Sagittal Index (mm)		45	70	55	65	70	65	50	75	35	70	65	60	50	45	70	100	65	75	65	70

MB: Milwaukee Brace, CSB: Chêneau–Sibilla Brace, SB: Sforzesco Brace; SSE: scoliosis-specific exercises; TR: treadmill FB: free body workout; ATR: angle of trunk rotation; TRACE: Trunk Aesthetic Clinical Evaluation Index; NP: not present. GH: Growth hormone. SD: Standard Deviation. Brace refers to the type of brace used in the period preceding the evaluation.

## Data Availability

The original contributions presented in the study are included in the article; further inquiries can be directed to the corresponding authors.

## Abbreviations

The following abbreviations are used in this manuscript:

PWS	Prader–Willi syndrome
SSE	Scoliosis-specific exercises
ATR	Angle of trunk rotation
TRACE	Trunk Aesthetic Clinical Evaluation
